# Anodal tDCS during face-name associations memory training in Alzheimer's patients

**DOI:** 10.3389/fnagi.2014.00038

**Published:** 2014-03-19

**Authors:** Maria Cotelli, Rosa Manenti, Michela Brambilla, Michela Petesi, Sandra Rosini, Clarissa Ferrari, Orazio Zanetti, Carlo Miniussi

**Affiliations:** ^1^IRCCS Centro San Giovanni di Dio - FatebenefratelliBrescia, Italy; ^2^Department of Clinical and Experimental Sciences, National Institute of Neuroscience, University of BresciaBrescia, Italy

**Keywords:** memory, stimulation, dementia, training, tDCS

## Abstract

**Objective:** Given the limited effectiveness of pharmacological treatments, non-pharmacological interventions to treat Alzheimer's disease (AD) have gained attention in recent years. The aim of the present study is to investigate the effects of anodal tDCS (AtDCS) combined with memory training on face-name associations in an AD patient sample.

**Methods:** Thirty six AD patients were randomly assigned to one of three study groups: *Group 1*, AtDCS plus individualized computerized memory training; *Group 2*, placebo tDCS plus individualized computerized memory training; *Group 3*, AtDCS plus motor training.

**Results:** A general improvement in performance was observed after 2 weeks of memory training. Both the anodal tDCS plus individualized computerized memory training and the placebo tDCS plus individualized computerized memory training groups had significantly improved performances at 2 weeks compared with the AtDCS plus motor training group.

**Conclusion:** Our findings suggest a beneficial effect of individualized memory rehabilitation in AD patients.

## Introduction

Memory impairment in patients with Alzheimer's disease (AD) is the core of this type of dementia. AD is a progressive disorder that affects several cognitive functions. However, some aspects of cognitive functions are preserved until later in the disease and can therefore be the targets of specific “rehabilitative/preservative” interventions (Clare et al., 2002; Werheid and Clare, 2007). The evidence regarding the neuropsychological profile of patients with AD justify the application of cognitive rehabilitation at an early dementia stage (Cotelli et al., 2012). Despite the memory deficits associated with AD, episodic memory can be enhanced when adequate support is provided (Backman and Dixon, 1992; Backman, 1996). Moreover, plasticity mechanisms also play a role in AD, since an increase in the activation of areas involved in memory or the recruitment of new areas has been previously shown (Becker et al., 1996; Woodard et al., 1998; Backman et al., 1999). However, cognitive rehabilitation aiming to improve memory function in dementia patients remains somewhat controversial (Clare et al., 2003b; Bahar-Fuchs et al., 2013).

Nevertheless, Clare and collaborators used a combination of methods to successfully produce long-lasting memories for a specific set of face-name pairs (Clare et al., 1999, 2000, 2001, 2002, 2003a). Furthermore, Davis et al. (2001) evaluated a 5-week cognitive intervention using face-name associations training, spaced retrieval, and cognitive stimulation in AD patients. Although the patients included in the intervention group showed improvements in face-name memory and in attention task, the beneficial results did not extend to other neuropsychological measures or to caregiver-rated patient quality of life. Interestingly, subsequent studies demonstrated that improvements in patient episodic memory remained stable 1 year after treatment (Clare et al., 2001).

Recently, in a study conducted by van Paasschen et al. (2013), fMRI was used to verify whether training-specific activations in people with early-stage AD occurred in the memory network during recognition of face-name pairs selectively in patients who received cognitive training. The findings showed training-specific increases in activation in the left middle and inferior frontal gyri, the left insula and the right medial parietal cortex.

Currently, there is growing interest in applying tDCS as an additional therapeutic approach in different disorders because its effects have been shown to outlast the stimulation period itself. In particular, anodal tDCS seems to be a good candidate to increase neuronal excitability and consequently performance in patients with cognitive deficits (Vallar and Bolognini, 2011).

tDCS generates an increase or a decrease in neuronal excitability that can modulate cognitive task performance by applying weak electrical currents directly to the head over a long period of time, usually on the order of minutes. tDCS delivers a weak polarizing electrical current to the cortex through a pair of electrodes, and brain excitability can be increased via anodal stimulation (AtDCS) or decreased via cathodal stimulation (CtDCS) depending on the polarity of the current flow (Nitsche et al., 2008; Paulus, 2011). Using this technique, short-term facilitation effects on cognitive functions in normal subjects and patients have been previously reported (Antal et al., 2004; Fregni et al., 2005; Monti et al., 2008; Nitsche et al., 2008; Sparing et al., 2009; Baker et al., 2010; Fertonani et al., 2010; Vallar and Bolognini, 2011; Manenti et al., 2013). It has also been shown that a single tDCS session can ameliorate memory deficits in AD patients (Ferrucci et al., 2008; Boggio et al., 2009).

Recently, Boggio et al. (2012) demonstrated that repeated sessions of anodal tDCS applied bilaterally over the temporal area led to an increase in performance of visual recognition memory tasks in a group of AD patients stable at a 4-week follow-up.

On the basis of the above data on the application of memory training in order to increase memory performance in AD patients and from preliminary data on the use of anodal tDCS in these patients, we hypothesized that a combined treatment could yield better results on memory performance in AD.

However, there are no studies to date that have explored the long-term effects of a combined treatment paradigm of tDCS during memory training to reduce or slow the cognitive decline in AD patients.

Therefore, the main purpose of the present study was to investigate whether the combined treatment of AtDCS applied to the left dorsolateral prefrontal cortex (DLPFC) and individualized computerized (IC) memory training would result in memory improvements in patients with AD. To address this question, we compared the effects of anodal or placebo tDCS combined with IC memory training vs. anodal tDCS combined with motor training on patient performance in a face-name association task (FNAT). Furthermore, we investigated whether the application of anodal tDCS could increase the effect of IC memory training. An increased improvement induced by anodal tDCS combined with IC memory training vs. placebo tDCS combined with IC memory training would support this hypothesis.

In addition, an important goal of the present study was to verify whether and for how long cognitive benefits might persist after the end of stimulation. Accordingly, we assessed the persistence of the effects three and 6 months after treatment.

## Materials and methods

### Participants

**Figure 1:**
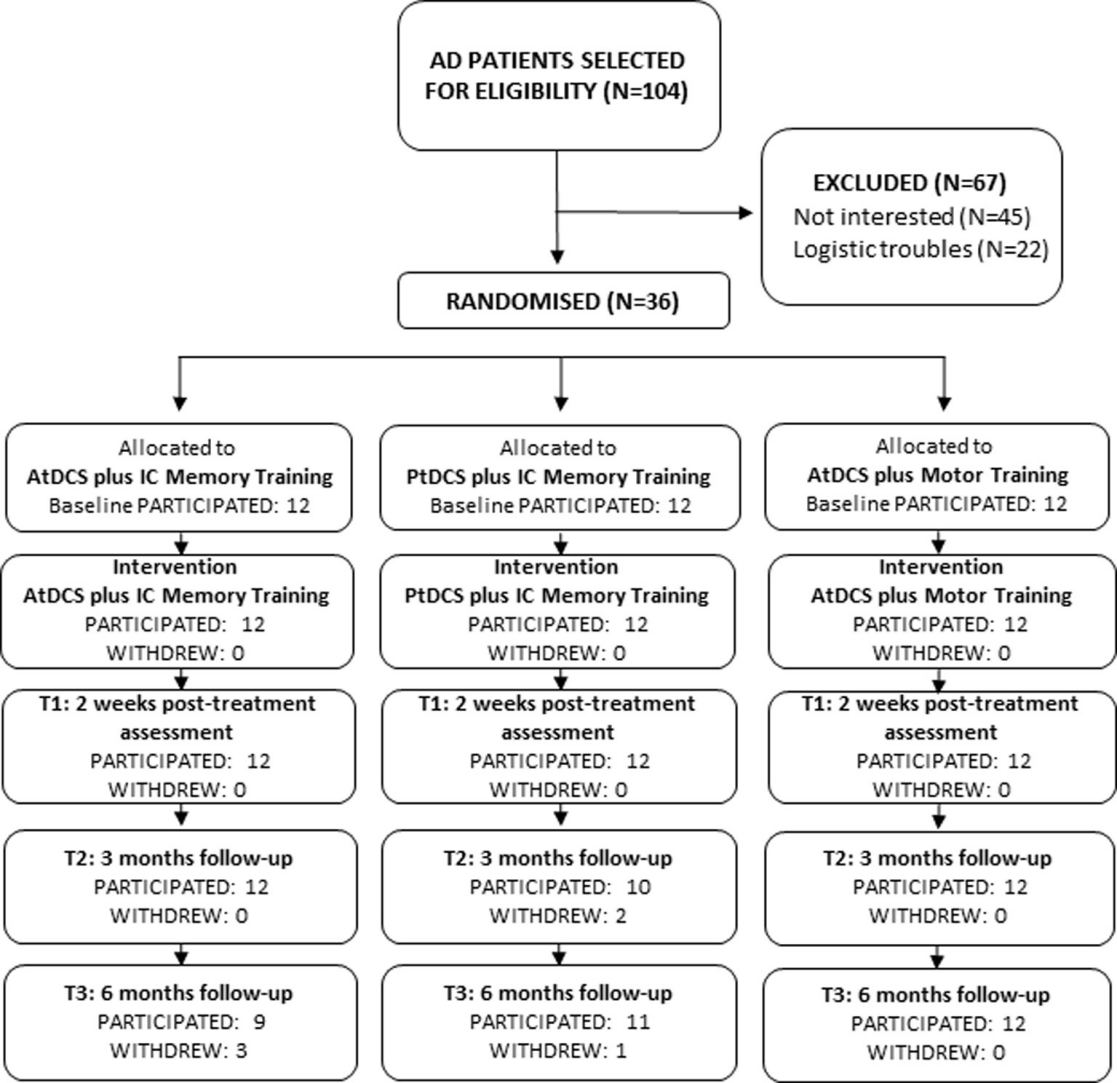
**Flow diagram of progression of participants through the study**.

Outpatients (*n* = 36) diagnosed as having probable mild to moderate AD, according to the NINCDS-ADRDA criteria (McKhann et al., 1984), were enrolled (Figure 1). Patients with potentially confounding neurological or psychiatric disorders, clinically recorded hearing or vision impairment were not included in the study. All patients had been on a stable dose of cholinesterase inhibitors (donepezil or rivastigmine) for at least 6 months prior to the onset of the study. All patients and caregivers signed and dated the Independent Ethics Committee/Institutional Review-approved written informed consent form before any study-specific assessment or procedure was performed.

### Study design

The 36 enrolled AD patients were randomly assigned to one of three treatment groups: Group 1—AtDCS plus IC Memory training (anodal tDCS during individualized computerized memory training); Group 2—PtDCS plus IC Memory training (placebo tDCS during individualized computerized memory training); and Group 3—AtDCS plus motor training (anodal tDCS during motor training).

**Figure 2:**
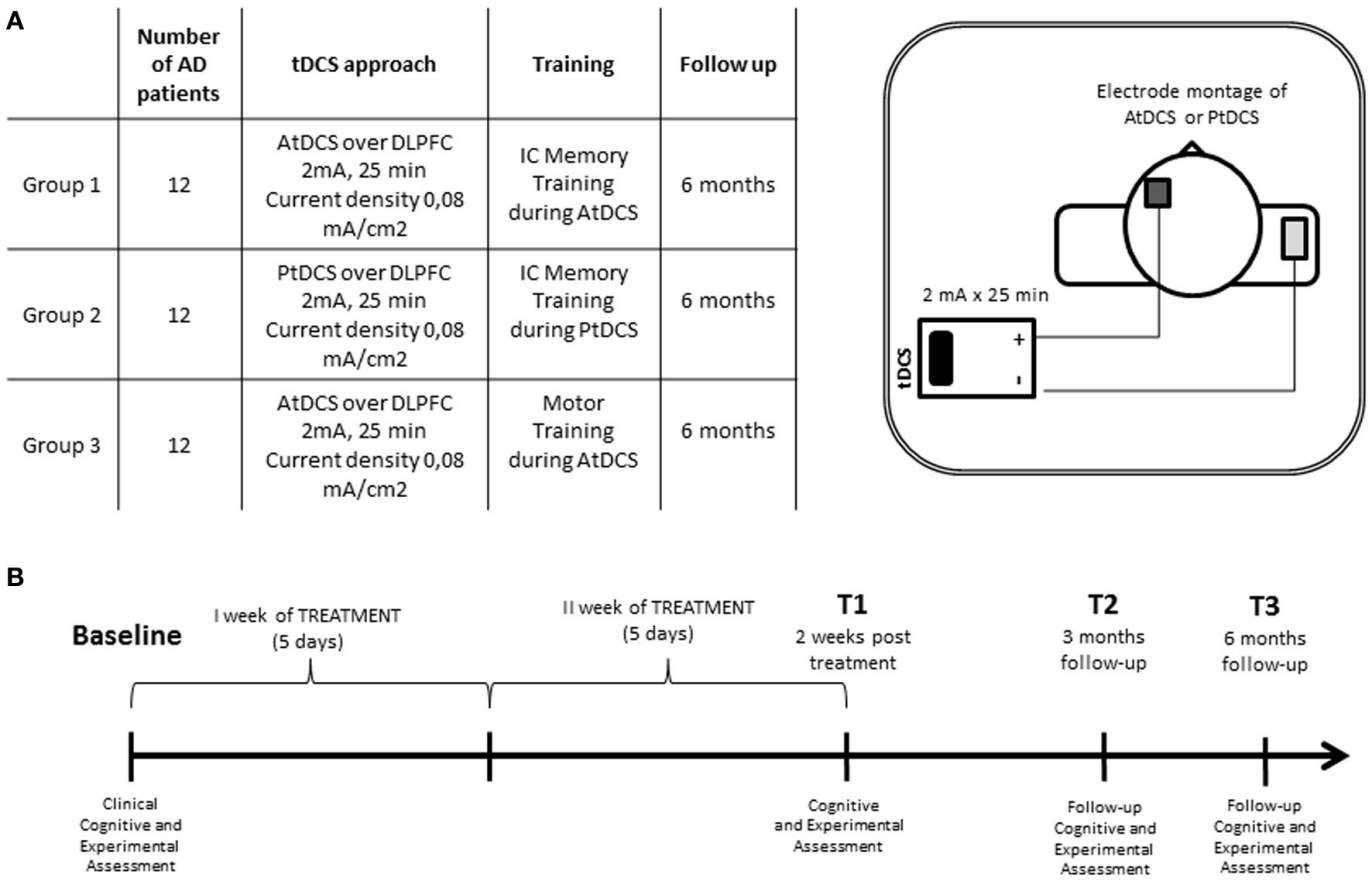
**(A)** Experimental conditions and electrode montage of AtDCS and PtDCS. **(B)** Experimental protocol of transcranial direct current stimulation combined with individualized computerized memory training.

The study was conducted in a double-blind manner to minimize potential bias from investigators and subjects. All patients underwent a neuropsychological and experimental assessment before (T0), after 2 weeks of treatment (T1), and 3 (T2) and 6 months (T3) after the beginning of treatment (Figure 2).

### Neuropsychological, functional and neuropsychiatric assessment

Two trained neuropsychologists, who were blinded to patient treatment allocations, administered the neuropsychological testing, divided into two sessions. All of the assessments (baseline, post-treatment and follow-ups) were administered for a single patient by the same assessor throughout the study.

The results of the cognitive, neuropsychiatric and functional assessments at baseline, before treatment (T0), and at 2 weeks (T1) and follow up (T2 and T3) are reported in Table 1 for the three experimental groups.

**Table 1:** **Demographic, neuropsychological, functional, and neuropsychiatric data analysis: mean scores, SD, and significance (*p*-value) of GLMM or LMM models**.

	**AtDCS plus memory training**				**PtDCS plus memory training**				**AtDCS plus motor**				**Cut-off**	***p*-value training (*n* = 12)**
**Age (years)**	**76.6 ± 4.6**				**74.7 ± 6.1**				**78.2 ± 5.2**				**–**	**ns**
**Education (years)**	**5.5 ± 2.4**				**8.9 ± 5.1**				**6.3 ± 2.6**				**–**	**ns**
**Gender (males/females)**	**2/10**				**3/9**				**2/10**				**–**	**ns**
	**Baseline**	**T1**	**T2**	**T3**	**Baseline**	**T1**	**T2**	**T3**	**Baseline**	**T1**	**T2**	**T3**		
**SCREENING FOR DEMENTIA**														
MMSE	20.1 ± 2.4	20.6 ± 2.4	19.7 ± 2.5	19.6 ± 3.5	20.8 ± 2.1	21.7 ± 3.5	20.5 ± 3.8	21.0 ± 2.5	22.1 ± 2.3	22.3 ± 2.4	21.0 ± 2.0	21.8 ± 2.3	>24	ns
**FUNCTIONAL STATUS**														
ADL	0.5 ± 1.2	0.5 ± 0.7	0.3 ± 0.7	0.4 ± 0.7	0.4 ± 0.7	0.5 ± 1.4	0.3 ± 0.5	0.7 ± 1.5	0.4 ± 0.7	0.2 ± 0.4	0.4 ± 0.5	0.6 ± 0.9	–	ns
IADL	3.7 ± 1.6	3.7 ± 1.4	3.9 ± 1.5	4.8 ± 1.6	2.3 ± 1.7	2.3 ± 1.5	2.8 ± 1.6	3.3 ± 2.5	3.8 ± 1.9	3.2 ± 1.7	3.7 ± 1.9	4.1 ± 1.8	–	ns
**BALANCE AND GAIT**														
Tinetti balance scale	13.9 ± 2.4	14.9 ± 2.0	14.5 ± 2.2	13.3 ± 3.6	15.0 ± 2.3	14.9 ± 2.3	15.1 ± 1.4	15.1 ± 1.4	14.3 ± 2.2	14.4 ± 2.1	14.3 ± 2.1	13.8 ± 3.4	–	ns
Tinetti gait scale	10.8 ± 2.3	10.9 ± 2.1	10.9 ± 2.3	10.3 ± 2.9	11.6 ± 0.8	11.4 ± 0.8	11.2 ± 1.3	10.9 ± 1.3	9.6 ± 3.1	10.1 ± 2.9	10.0 ± 3.1	10.1 ± 2.9	–	ns
**BEHAVIORAL SYMPTOMS**														
NPI	13.3 ± 13.8	13.9 ± 13.0	15.0 ± 13.8	16.0 ± 9.0	14.1 ± 12.5	14.3 ± 15.5	15.7 ± 17.6	13.5 ± 11.0	7.75 ± 3.4	5.8 ± 4.1	6.3 ± 4.5	8.6 ± 4.9	–	ns
**LANGUAGE**														
**Picture naming task**														
Objects (correct responses, %)	65.8 ± 15.6	68.4 ± 17.7	61.3 ± 19.5	70.9 ± 12.1	68.6 ± 24.0	70.3 ± 24.6	72.8 ± 13.8	73.4 ± 15.7	77.0 ± 18.1	79.4 ± 18.3	80.1 ± 19.2	82.7 ± 12.7	–	ns
Actions (correct responses, %)	50.3 ± 17.7	53.8 ± 19.9	53.1 ± 20.9	59.6 ± 14.6	54.4 ± 25.5	56.8 ± 23.9	61.2 ± 20.5	62.0 ± 13.9	66.5 ± 24.1	69.1 ± 24.4	65.4 ± 22.9	70.0 ± 17.1	–	ns
**BADA (correct responses, %)**														
Oral object naming	61.3 ± 23.4	64.3 ± 22.3	64.2 ± 19.9	65.2 ± 15.7	63.7 ± 25.0	66.1 ± 25.3	68.1 ± 21.3	67.7 ± 21.5	74.5 ± 21.3	77.6 ± 22.1	77.6 ± 23.3	80.0 ± 15.7	–	ns
Oral action naming	56.1 ± 22.2	58.5 ± 21.7	57.6 ± 21.3	61.0 ± 15.3	59.3 ± 23.6	60.4 ± 24.9	60.4 ± 18.3	63.0 ± 16.5	67.5 ± 24.6	68.8 ± 23.5	69.9 ± 25.8	72.7 ± 18.5	–	ns
Sentence comprehension	79.0 ± 9.1	78.9 ± 11.6	75.5 ± 11.4	79.6 ± 11.9	82.2 ± 9.7	87.0 ± 8.4	86.6 ± 8.9	82.4 ± 13.5	85.3 ± 10.6	86.7 ± 9.5	87.7 ± 14.2	90.5 ± 7.2	–	ns
**MEMORY**														
**Rivermead behavioral memory test**														
Story recall-immediate	0.9 ± 0.9	1.5 ± 1.3	0.8 ± 0.8	1.4 ± 1.4	1.4 ± 1.0	1.4 ± 0.8	1.7 ± 1.5	1.5 ± 1.5	0.5 ± 0.6	0.8 ± 0.8	0.5 ± 0.6	0.7 ± 0.9	>6	ns
Story recall-delayed	0.1 ± 0.3	0.6 ± 1.0	0.1 ± 0.3	0.5 ± 1.5	0.4 ± 0.7	0.7 ± 1.0	1.2 ± 2.2	0.6 ± 1.0	0.0 ± 0.0	0.3 ± 1.0	0.0 ± 0.0	0.0 ± 0.0	>4	ns
**Rey auditory verbal learning test**														
Immediate recall	18.0 ± 8.2	19.3 ± 9.1	19.8 ± 8.9	20.7 ± 8.4	18.5 ± 7.3	20.6 ± 9.4	19.8 ± 8.1	16.8 ± 5.8	17.2 ± 4.2	18.9 ± 4.4	19.0 ± 3.7	16.7 ± 5.5	>28.52	ns
Delayed recall	1.3 ± 1.4	2.5 ± 1.7	2.4 ± 2.0	2.7 ± 1.9	1.9 ± 1.9	2.1 ± 2.4	2.1 ± 1.7	1.3 ± 1.5	0.3 ± 0.6	0.5 ± 0.8	0.4 ± 0.9	0.3 ± 0.6	>4.68	ns
Rey-Osterrieth Complex figure-recall	0.5 ± 1.6	0.8 ± 1.9	0.1 ± 0.1	0.2 ± 0.7	1.1 ± 3.1	1.2 ± 2.6	1.2 ± 1.7	0.6 ± 1.5	0.1 ± 0.3	0.2 ± 0.4	0.1 ± 0.4	0.3 ± 0.5	>9.46	ns
**PRAXIS**														
Rey-osterrieth Complex figure-copy	11.9 ± 11.0	16.7 ± 10.6	12.5 ± 10.3	12.9 ± 9.4	17.6 ± 10.6	18.6 ± 10.3	15.6 ± 11.2	15.1 ± 11.4	16.9 ± 10.8	16.8 ± 11.4	15.3 ± 9.3	18.7 ± 11.7	>28.87	ns
**ATTENTIONAL AND EXECUTIVE FUNCTIONS**														
Trail making test-A	231 ± 137	190 ± 115	187 ± 101	164 ± 100	135 ± 51	120 ± 45	135 ± 53	129 ± 54	129 ± 82	134 ± 82	118 ± 77	126 ± 72	<93	0.015^*^
Trail making test-B	551 ± 108	536 ± 136	554 ± 106	566 ± 114	431 ± 103	437 ± 99	400 ± 140	440 ± 91	445 ± 96	437 ± 105	438 ± 107	463 ± 63	<282	ns

*T0, Baseline assessment; T1, post-treatment assessment; T2, 3 months follow-up assessment; T3, 6 months follow-up assessment; MMSE, Mini Mental State Examination; ADL, Activities of daily living; IADL, Instrumental activities of daily living; NPI, Neuropsychiatric Inventory; BADA, Battery for Analysis of Aphasic Deficits; ns, not significant. Cut-off scores according to Italian normative data are reported. Raw scores are reported*.

* *AtDCS plus memory training and PtDCS plus memory training improved their performance over time and are better than AtDCS plus motor training at 24 weeks*.

### Experimental evaluation: face-name association memory task (FNAT)

The Face-Name Association memory Task (FNAT) was used to assess the patient's associative memory and was composed of encoding and retrieval phases.

Subjects were seated in a dimly lit room, facing a computer monitor that was placed 60 cm from the subject. The stimuli were presented using Presentation software (Version 14.9, www.neurobs.com) running on a personal computer with a 15-inch screen. Verbal responses were recorded and digitized at 44.1 kHz using GoldWave (V. 5.12, www.goldwave.com).

During the encoding phase, the patient was shown a gray-scale picture of a face on a monitor together with a proper name, and the patient was required to tell the researcher whether the face belonged to a woman or a man and was required to encode the face-name association. A set of 60 unfamiliar faces associated to a set of 60 unfamiliar proper names (30 male, 30 female). During the retrieval phase, the patient was shown a face together with four proper names (the correct name, two previously presented names and one new name), and the patient was asked to associate the correct name with each face.

All 36 enrolled AD patients underwent the FNAT before treatment (T0), at 2 weeks (T1) and at follow ups (T2 and T3).

### Individualized computerized (IC) memory training

The memory training protocol was developed based on the individualized performance of each patient in the FNAT. For each subject, we selected 40 face-name pairs that were incorrectly retrieved and randomly assigned the face-name pairs to treated and untreated (control) lists, which were each composed of 20 stimuli. Accordingly, 20 face-name pairs were shown in total to each subject during the 2 weeks of FNAT training.

All patients assigned to IC memory training underwent a daily therapy session 5 days per week (i.e., from Monday to Friday). For each patient, an individual training experiment was created to individually separate the treated and control lists. Over 10 days of training, 20 face-name pairs were learned (i.e., 2 face-name pairs per daily session and 10 face-name pairs per week). The treatment was based on an errorless approach in which the participants were encouraged not to guess but to respond only when they were sure of the correct answer during all the sessions.

The daily session included the following steps:

1. *Encoding*—After the presentation of the face-name pair, each participant was asked to choose if the face shown was a male or a female and to try to estimate the age.
2. *Vanishing cues*—Each participant was presented with the correct face-name pair, minus the last letter of the name, thus ensuring a correct recall. They were then presented with the name minus the last two letters, and so on, until the patients could recall the name when no letter cues were presented.
3. *Visual mnemonics*—The patient's attention was directed to three distinctive visual features of the face to be remembered (established *a priori* for each face) and were asked to create a mental image of these visual features and link them to the correct name.
4. *Expanding rehearsal*—Once the face-name association was learned using the methods above, the patient was asked to recall the name after increasingly long intervals: initially 30 s and then 1, 2, and 5 min (Clare et al., 1999).
5. *Review*—The training sessions ended with a review of the two face-name pairs trained during the daily session.

The sessions following the first session started with a review of all of the face-name pairs learned on previous days.

Following the final session of the 10-day training period, the participants were tested using a FNAT (as at baseline) including both the trained and untrained lists.

### Motor training

We established a standardized sequence of motor exercises. All patients assigned to motor training underwent a daily therapy session 5 days per week (i.e., from Monday to Friday). The motor training program was conducted by a physiotherapist and was divided into two phases: Step 1—walking rehabilitation and Step 2—balance and coordination exercises. Each step included 6 exercises each lasting approximately 90 s, and four breaks were included at fixed time-points. Step 1 started with three exercises within a ring; next, two exercises were carried out with a step. Finally, patients were asked to cycle for 120 s followed by a third break, which concluded Step 1. Step 2 consisted of six exercises (without tools) divided into two subgroups with a break between (1) raising one's shoulders in an alternating manner; (2) alternately touching the floor with the tips of one's toes and one's heels; (3) extending the left and right legs in an alternating manner; (4) making circles on the floor with the left and right legs with 1 s of rest between each circle; (5) turning one's wrists; and (6) opening and closing one's hands.

### tDCS

All of the patients received 2 weeks of tDCS stimulation over the left DLPFC. Each week of tDCS treatment consisted of 5 sessions of 25 min/day starting from the beginning of the specific training (IC memory or motor training). The stimulation was delivered using a battery-driven constant-current stimulator (BrainStim, EMS, Bologna, Italy) through a pair of saline-soaked sponge electrodes. The active electrode (5 × 5 cm) was placed on the left DLPFC, 8 cm frontally and 6 cm laterally with respect to the scalp vertex. The reference electrode (6 × 10 cm) was placed on the right deltoid muscle.

A constant current of 2 mA (current density 0.08 mA/cm^2^) was applied with a ramping period of 10 s at the beginning and end of the stimulation (Poreisz et al., 2007; Nitsche et al., 2008; Nitsche and Paulus, 2011). The current density of the active electrode was maintained below the safety limits (Poreisz et al., 2007; Nitsche et al., 2008). In the sham stimulation (i.e., placebo), the current was turned off 10 s after the beginning of the stimulation (plus the duration of the fade-in = 10) and was turned on for the last 10 s of the stimulation period (plus the duration of the fade-out = 10), making this condition indistinguishable from the experimental stimulation.

### Statistical analysis

Statistical analyses were performed using Statistica software (version 10; www.statsoft.com) and R language and environmental for statistical computing version 2.15.1. (R Development Core Team, 2011).

The homogeneity of the cognitive assessments at baseline among the three experimental groups was evaluated by a univariate ANOVA model and, for variables violating Shapiro-Wilk normality test, by a non-parametric Kruskal-Wallis test.

Generalized linear mixed models (GLMM- fitted by the Laplace approximation method) (Breslow and Clayton, 1993) for repeated measures (*time* as within factor) were adopted for analyzing the non-Normal (Binomially distributed) dependent variables for the FNAT experiment, including the *group* variable as a between factor. The same GLMMs for Binomial data were performed to analyze data from the International Picture Naming Task, and for naming and sentence comprehension data from the Battery for Analysis of Aphasic Deficits (BADA) and the Rey Auditory Verbal Learning Test. For the rest of the cognitive assessment tools (non-Binomial distributed), linear Mixed Models for repeated measures with the *group* variable as the between factor were adopted.

Bonferroni corrections were adopted for all comparison adjustments of *post-hoc* analyses. Statistical significance was set at *p* < 0.05.

## Results

No differences in age and education were observed between the three groups (ANOVA for age as dependent variable: *F* = 1.22, *p* = 0.309; ANOVA for education -years- as dependent variable: *F* = 3.01, *p* = 0.063). Moreover, no differences were detected among the three groups at baseline for the FNAT experiments (Kruskal–Wallis *p* = 0.350) as well as for all cognitive assessment tools.

### FNAT data

Two different GLMMs were applied to treated and untreated subjects. For the former, a general improvement in performance was observed after 2 weeks of memory training (T1 vs. T0 time: *z* = 3.14, *p* = 0.002); the groups AtDCS plus IC memory training and PtDCS plus IC memory training showed significantly improved performances compared with the AtDCS plus motor training group after 2 weeks of treatment (AtDCS plus IC memory training vs. AtDCS plus motor training: *z* = 3.67, *p* < 0.001, PtDCS plus IC memory training vs. AtDCS plus motor training: *z* = 3.08, *p* = 0.002). Both the AtDCS plus IC memory training and the PtDCS plus IC memory training groups maintained similar performances across time by not highlighting any differences (AtDCS plus IC memory training vs. PtDCS plus IC memory training: *z* = −0.05, *p* = 0.646).

Moreover, the effect of memory training on the PtDCS plus IC memory training group compared with the AtDCS plus motor training group was still significant after 12 weeks (PtDCS plus IC memory training vs. AtDCS plus motor training: *z* = 2.29, *p* = 0.021). No significant effects for the untreated face-name pairs were observed for the AtDCS plus IC memory training group compared with the AtDCS plus motor training group after 12 weeks (*z* = 1.59, *p* = 0.111).

**Figure 3:**
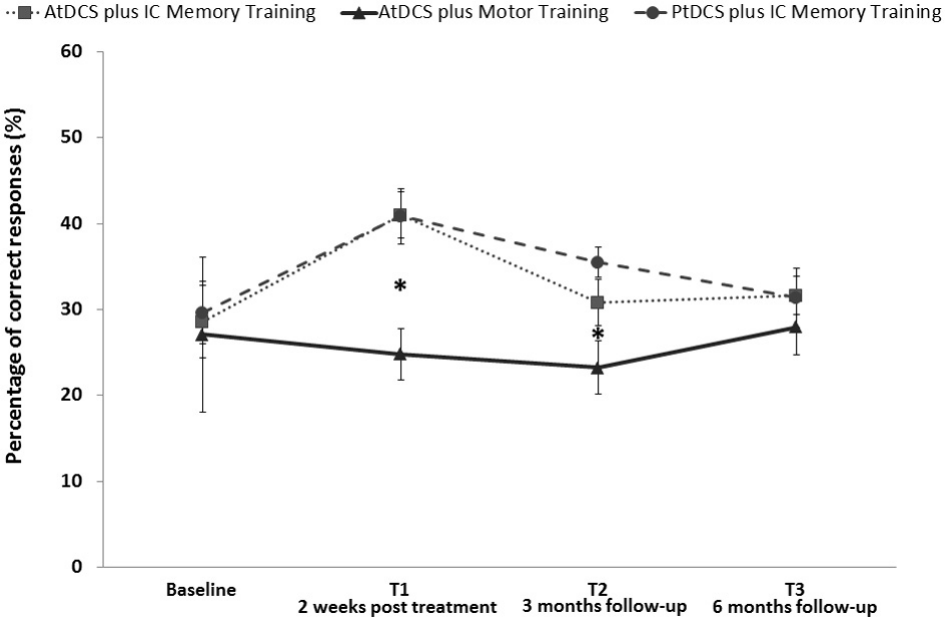
Face-Name associations task (FNAT) memory accuracy (%) for treated stimuli at baseline (T0), after 2 weeks of treatment (T1) and 3 (T2) and 6 months (T3) after the beginning in AD patients who received AtDCS plus IC memory training, PtDCS plus IC memory training or AtDCS plus motor training (^*^p < 0.05). Error bars represent standard errors. Asterisks indicate the significant improvement in FNAT performance induced by AtDCS plus IC memory training and PtDCS plus IC memory training in comparison to AtDCS plus motor training at T1 and T2 evaluation.

No significant for the non-treated face-name pairs effects were observed (Figure 3).

### Cognitive instruments

No effects of tDCS plus IC memory training on neuropsychological instruments and functional scales were detected. However, we observed an improvement in the performance of the AtDCS plus IC memory training group and the PtDCS plus IC memory training group after 6 months (T3) in the Trail Making Test part A score (*t* = −5.05, *p* = 0.015).

## Discussion

The main purpose of this study was to investigate whether the application of combined AtDCS to the left DLFPC plus individualized computerized memory training for 25 min a day, 5 days a week, for 2 weeks would lead to significant memory improvements in patients with AD. Specifically, we hypothesized that this protocol would result in a facilitation of performance in a face-name association task (FNAT). To address this question, we compared the effect of AtDCS or PtDCS combined with memory training with AtDCS combined with motor training on the performance in a face-name association task. Moreover, by directly comparing the two IC memory training groups, we aimed to observe additional gain induced by AtDCS.

Another important aim of the present study was to verify whether the cognitive benefits recorded immediately after treatment would persist for three and 6 months after the treatment protocol.

Overall, the results of our study showed a significant improvement in face-name association task performance, selectively for trained stimuli, induced by individualized computerized memory training irrespective of the tDCS protocol. AD patients who received a memory intervention (the AtDCS plus IC memory training and PtDCS plus IC memory training groups) showed gains in performance of trained stimuli of a face-name association task compared with patients who received AtDCS plus motor training. The effects of the applied combined treatment have been recorded only for FNAT trained stimuli and did not generalize to control stimuli or to other learning and memory tasks highlighting the specific effect of the memory training.

Importantly, in contrast with previous studies that applied tDCS in AD (Ferrucci et al., 2008; Boggio et al., 2009, 2012), in the present work, we failed to observe a significant additional effect of AtDCS on memory performance in AD. Combined treatment (anodal tDCS plus IC memory training) did not ameliorate the memory performance associated with AD more than memory treatment alone (placebo tDCS during IC memory training).

This lack of an effect might be related to the different tDCS protocol used in previous studies. In the present study, we used a tDCS approach in which patients received daily tDCS treatment combined with IC memory training or motor training, while in previous studies a single session or repeated sessions of tDCS alone were applied. To the best of our knowledge, the present study is the first that applied combined tDCS and memory training in AD patients. Moreover, previously reported enhancements in memory following anodal tDCS in AD patients concerned memory processes that involved a single stimulus, for instance working memory and verbal or visual recognition memory of images or words (Ferrucci et al., 2008; Boggio et al., 2009, 2012). Conversely, in the present report, we assessed associative memory (face and name) processing. Testing memory for face-name associations provides a significant measurement of episodic and semantic memory (Werheid and Clare, 2007). Furthermore, during memory rehabilitation interventions, we applied an errorless learning method that employs learning conditions in which patients are prevented from making errors (e.g., Vanishing cues and expanding rehearsal). Hammer et al. (2011) found that anodal tDCS applied over the left DLPFC did not modulate memory performance following errorless or errorful learning in young healthy participants, which seems to be in accordance with the present findings.

The present result suggests that sometimes non-additive mechanisms might be present combining two “plasticity” inducing protocols. Namely a homeostatic mechanism that is activated during “high” excitability levels (i.e., increase excitability induced by learning plus increased excitability induced by AtDCS) to keep the system within a normal functional range. In this respect homeostatic plasticity has been previously shown to block overnight consolidation of learning after AtDCS (Peters et al., 2013). This might explain why after 12 weeks improvement in performance was still significant only for the placebo tDCS memory training group.

Additionally a substantial body of research has shown that tDCS induces modifications of cortical plasticity that may outlast the stimulation period itself (Dayan et al., 2013). Nevertheless, the mechanisms underlying the effects of tDCS on memory and learning are not yet understood, and may involve changes in the neuromodulation efficacy of different neurotransmitters (Clark and Parasuraman, 2013; Coffman et al., 2013; Dayan et al., 2013). In AD patients, anodal tDCS has been applied to increase cortical activity, as AD patients show temporo-parietal hypoactivity (Fernandez et al., 2002). The opposite approach, cathodal tDCS to reduce hyperexcitability in frontal areas, may have beneficial effects in AD (Hansen, 2012). Taken together, these data suggest that stimulation techniques appear safe in AD patients, but the precise short and long-term effects have not been sufficiently evaluated (Freitas et al., 2011). Further studies are needed to identify the optimal responders to specific non-invasive brain stimulation interventions (Boggio et al., 2011; Guerra et al., 2011; Vallar and Bolognini, 2011), and more research is needed to better understand how tDCS in combination with a cognitive task works (de Berker et al., 2013).

We identified an improvement in the face-name association memory task 3 months after the intervention only in AD patients who received PtDCS plus IC memory training compared with patients who received AtDCS plus motor training. Moreover, both AD groups that received memory training maintained similar performances across all time points.

Our results are in line with previous studies that highlight that cognitive interventions can have therapeutic benefits in patients with AD (Buschert et al., 2011). However, cognitive rehabilitation and cognitive training focusing on memory functioning in dementia patients remains somewhat controversial (Clare et al., 2003b; Cotelli et al., 2006; Bahar-Fuchs et al., 2013).

Several limitations to this pilot study need to be acknowledged. The relatively small number of patients, the lack of a placebo stimulation group without any treatment, and a longer follow-up required to evaluate the trajectories of progression represent some limitations. A longer follow-up time would clarify whether additional rehabilitation protocols should be considered over time. Moreover, anodal and placebo stimulation over the left DLPFC with an extracephalic reference site was used. Cathodal stimulation might induce beneficial effects in these patients (Hansen, 2012). Other cerebral areas (e.g., temporal) or different reference site (e.g., cephalic) could also be tested.

Despite these limitations of our tDCS experiments, the robust behavioral changes observed in the memory task are quite encouraging and should serve as the basis for future studies. Further studies, based on larger patient samples and including placebo and control conditions, should be conducted to identify the optimal parameters for a combined treatment protocol. The development of uniform protocols is necessary to allow a direct comparison between the studies (Brasil-Neto, 2012), and more research is needed to identify which patients would be the optimal responders to a combined treatment protocol. Moreover, further lines of inquiry should evaluate the functional changes in cortical reactivity and effective connectivity induced by these protocols.

In summary, a general improvement in performance was observed after 2 weeks of individualized computerized memory training irrespective of the tDCS protocol (placebo vs. real). Moreover, such effect was still significant after 12 weeks but only for the placebo stimulation. Although further controlled studies are needed to demonstrate the efficacy of cognitive training and stimulation interventions, the current pilot study highlights that an individualized computerized memory treatment might be useful in enhancing memory functioning in AD patients, and that anodal tDCS effects may not be always additive during a memory rehabilitation protocol.

### Conflict of interest statement

The authors declare that the research was conducted in the absence of any commercial or financial relationships that could be construed as a potential conflict of interest.
